# The BDNF/TrkB Neurotrophin System in the Sensory Organs of Zebrafish

**DOI:** 10.3390/ijms23052621

**Published:** 2022-02-27

**Authors:** Marialuisa Aragona, Caterina Porcino, Maria Cristina Guerrera, Giuseppe Montalbano, Rosaria Laurà, Marzio Cometa, Maria Levanti, Francesco Abbate, Teresa Cobo, Gabriel Capitelli, José A. Vega, Antonino Germanà

**Affiliations:** 1 Zebrafish Neuromorphology Lab, Department of Veterinary Sciences, University of Messina, 98168 Messina, Italy; mlaragona@unime.it (M.A.); catporcino@unime.it (C.P.); mguerrera@unime.it (M.C.G.); gmontalbano@unime.it (G.M.); laurar@unime.it (R.L.); marzio.cometa@unime.it (M.C.); mblevanti@unime.it (M.L.); abbatef@unime.it (F.A.); 2 Departamento de Cirugía y Especialidades Médico-Quirúrgicas, Universidad de Oviedo, 33006 Oviedo, Spain; tcobo@uniovi.es; 3 Faculty of Medical Sciences, University of Buenos Aires, Viamonte 1053, CABA, Buenos Aires 1056, Argentina; gcapitelli@rec.uba.ar; 4 Grupo SINPOS, Universidad de Oviedo, 33003 Oviedo, Spain; javega@uniovi.es; 5 Departamento de Morfología y Biología Celular, Universidad de Oviedo, 33006 Oviedo, Spain; 6 Facultad de Ciencias de la Salud, Universidad Autónoma de Chile, Santiago 7500912, Chile

**Keywords:** BDNF, TrkB receptor, neurotrophins, zebrafish, sensory systems

## Abstract

The brain-derived neurotrophic factor (BDNF) was discovered in the last century, and identified as a member of the neurotrophin family. BDNF shares approximately 50% of its amino acid with other neurotrophins such as NGF, NT-3 and NT-4/5, and its linear amino acid sequences in zebrafish (*Danio rerio*) and human are 91% identical. BDNF functions can be mediated by two categories of receptors: p75NTR and Trk. Intriguingly, BDNF receptors were highly conserved in the process of evolution, as were the other NTs’ receptors. In this review, we update current knowledge about the distribution and functions of the BDNF-TrkB system in the sensory organs of zebrafish. In fish, particularly in zebrafish, the distribution and functions of BDNF and TrkB in the brain have been widely studied. Both components of the system, associated or segregated, are also present outside the central nervous system, especially in sensory organs including the inner ear, lateral line system, retina, taste buds and olfactory epithelium.

## 1. Introduction

### 1.1. Neurotrophins Signaling System

The brain-derived neurotrophic factor (BDNF) was discovered by Barde et al. [1], and identified as a member of the neurotrophin family, which also includes nerve growth factor (NGF), neurotrophin (NT) 3, and NT 4/5 [2]. Two orthologs of NGF, denominated NT-6 [3] and NT-7 [4,5], have also been identified in teleosts. Neurotrophins are evolutionarily conserved and have been found in both fish and in mammals [6,7,8,9,10,11,12,13]. In particular, *bdnf* encodes for proBDNF, that, in turn, progressively cleaves to generate the mature BDNF [14]. BDNF shares approximately 50% of its amino acid with NGF, NT-3, and NT-4/5 [15], and the primary amino acid sequences of zebrafish and human BDNF are 91% identical [16,17]. Interestingly, as for NTs, the signaling receptors are highly conserved within evolution [18,19,20,21]. As for the other neurotrophins, BDNF physiological functions are mediated by two classes of receptors, p75NTR and Trk [22]. NGF binds to TrkA, BDNF, and NT-4 to TrkB, and NT-3 to TrkC, but even if neurotrophins bind specifically to their own receptor, they can also interact with the other receptors. NT-3, for instance, can also bind to TrkA and TrkB and all neurotrophins can generally bind to p75 receptors. Within this system, a fine control of ligand–receptor interactions are allowed by the association of p75 with Trk receptors regulating the affinity of Trk receptors for each neurotrophin [23,24,25,26,27]. After ligand binding, Trk receptors are activated by a dimerization causing an autophosphorylation of specific tyrosine residues leading to the initiation of the signaling system. This latter involves tyrosine kinase substrates interacting with key docking sites on Trk receptors. This interaction has been proposed to facilitate interactions with ion channels, and to indirectly activate the Ras/MAPK (mitogen-activated protein kinase) [28]. Mature BDNF can bind to both full-length and truncated TrkB receptor isoforms. The full-length isoform modulates signaling pathway via an intracellular kinase domain while the truncated TrkB, without kinase domain, binds and internalizes BDNF, causing its inhibition. In teleost fish, the TrkB receptor is present as *TrkB1* and *TrkB2,* due to a specific genome duplication, and the amino acid residues of its kinase domain are identical in zebrafish and mammals [29,30,31]. In zebrafish, NGF, NT3, and NT4 have been detected, respectively, in the brain and the cerebellum of adults and during development [32,33,34]. In particular, the distribution and functions of BDNF and TrkB in the brain have been widely studied [26,27,28,29,30,31,32]. The investigation of BDNF functions, in earlier vertebrates than mammals, aims to provide additional and clearer information on BDNF physiological functions on the neurophysiology of the brain [35]. Both BDNF and TrkB, associated or segregated, are also present outside the central nervous system, especially in sensory organs including the inner ear [33], lateral line system [34], retina [35,36], taste buds [37], and olfactory epithelium.

### 1.2. Zebrafish and Its Application on Sensory Organs

Zebrafish have become a prominent vertebrate model for studying diseases [36,37] since they have 82% orthologues of human disease-associated genes [37]. Recent evidence affirms that zebrafish represent a novel suitable model to study Alzheimer’s disease [38], Huntington’s disease [39], traumatic brain injury [32,40], and sensory organs’ diseases, especially blindness and deafness. Among factors involved in those pathologies is the BDNF/TrkB axis. However, even though its contribution in stimulating tissue repair after brain lesions has been demonstrated [41], its role in the sensory organs of zebrafish is still poorly understood, and most of the suspected function must be extrapolated from experiments in mammals. The sensory system in fish consists of specialized sensory organs that contain differentiated cells able to detect light, mechanical, and chemical environmental stimuli, and convert them into electric signals. For all these reasons, zebrafish are employed as a model to study different human sensory disorders. Zebrafish have been established as a valuable model for the study of the development and regeneration of retinal cells, [42] and have been largely used as a model in ophthalmology [43]. Genetic screens in zebrafish helped, not only to establish additional genetic models for photoreceptor degenerations but also to identify additional genes involved in retinal degeneration. However, remarkable differences exist with mammals. In fact, in mammals, the retina does not show a regeneration phenomenon after damage, and in contrast the zebrafish retina has the capacity for regeneration due to its continued growth into adulthood and the ability of this fish model to maintain a population of multipotent stem cells [44,45]. While the effects of BDNF in the protection of retinal ganglion cells are evident, the therapeutic use of BDNF in chronic models of glaucoma is still at the stage of preliminary experiments [46]. In the inner ear, BDNF may act alone or in cooperation with other neurotrophins to establish the afferent innervation of the inner ear sensory epithelium [47]. In point of fact, mice lacking either BDNF or its associated receptor, TrkB, lose innervation to the semicircular canals and have reduced innervation of the outer hair cells in the apical and middle turns of the cochlea [48]. During cochlear development, neurotrophins regulate neuronal differentiation and survival [49,50]. Consequently, there has been great interest in recent years in exploring potential therapies based on BDNF to ameliorate the degeneration of the cochlear ganglion neurons after deafness. Neurotrophic support to the cochlear is provided by hair cells, the supporting cells of the organ of Corti, and the neurons of the cochlear nucleus [51]. The aforementioned physiological functions of BDNF and TrkB were reported as well in human cochlear ganglion neurons [52]. Numerous studies have demonstrated that exogenous BDNF delivered directly to the cochlea can protect cochlear neurons and promote neuronal survival after deafness [53,54,55]. BDNF is required for the normal development of taste neurons [56] and the gustatory papillae and taste buds [57], as well as for maintenance of normal innervation [58]. Taste bud cells die and are replaced continuously in adulthood, and new taste bud cells must be innervated by nerve fibers. In this process, BDNF plays a crucial part. [58,59,60]. BDNF has an important role in both olfactory epithelium and olfactory bulb regeneration after injury [61]. Therefore, given the importance of the BDNF-TrkB system in the physiopathology of sensory organs, in this review, we updated the current knowledge about its distribution and functions in the sensory organs of zebrafish.

## 2. BDNF-TrkB and Hair Cells: The Lateral Line System and Inner Ear

**Figure 1:**
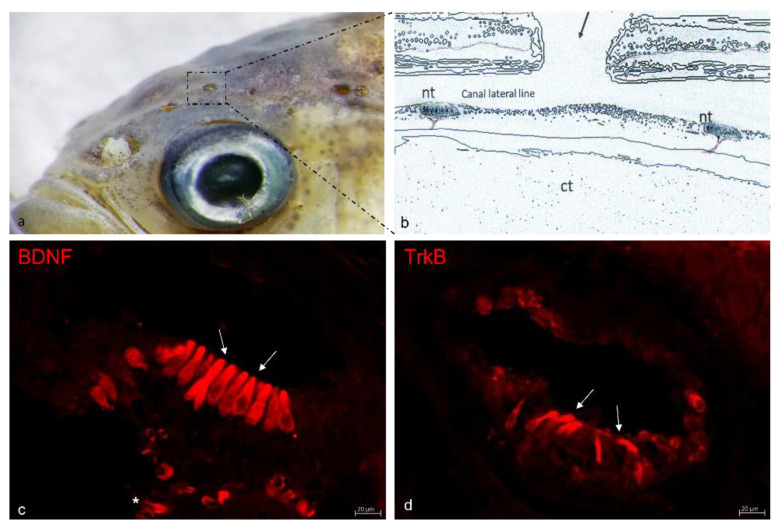
Stereomicrograph of zebrafish head with lateral line system canal pore (**a**). Graphical representation of the canal of the lateral line system; pore (arrow), neuromast (nt) and connettive tissue (ct); (**b**). Immunohistochemical detection of BDNF; (**c**) and TrkB; (**d**) in the sensory hair cells (white arrows) of supra-orbital neuromasts. Magnification 40× (**c**,**d**).

The octavolateralis system in zebrafish is formed of the lateral line system and the inner ear. This system plays a fundamental role in the mechano-detection of the external stimulus. The lateral line is the major mechanosensory system in fish [62,63] consisting of a cluster of sensory hair cells with basal, supporting and mantle cells forming the so-called neuromasts which are localized both in the skin surface (free neuromasts) and in the bony canals of the head and body (deep neuromasts) [64]. The neuromast innervation consists, as in other sensory organs, of afferent and efferent nerve fibers connecting the peripheral hair cells with the brain. Regarding the efferent innervation, three nuclei have been identified in the brain and localized two in the hindbrain and one in the ventral hypothalamus, while in the afferent part the sensory hair cells are connected to the cephalic and posterior ganglion neurons [65,66]. The sensory hair cells of the lateral line system allow fish to detect weak water motions, pressure gradients, balance, and orientation [67]. Furthermore, they participate in predator avoidance, prey capture, schooling, and mating [68]. The occurrence of in situ hybridization for BDNF transcripts in the hair cells of the zebrafish neuromasts was reported for the first time in 4-day-old larvae [16]. Then, using real-time quantitative PCR and immunohistochemistry for detection of BDNF and TrkB in the lateral line, the occurrence of both components of the system was studied in animals from 10 to 180 days post-fertilization (dpf) in wild-type zebrafish (Figure 1).

**Figure 2:**
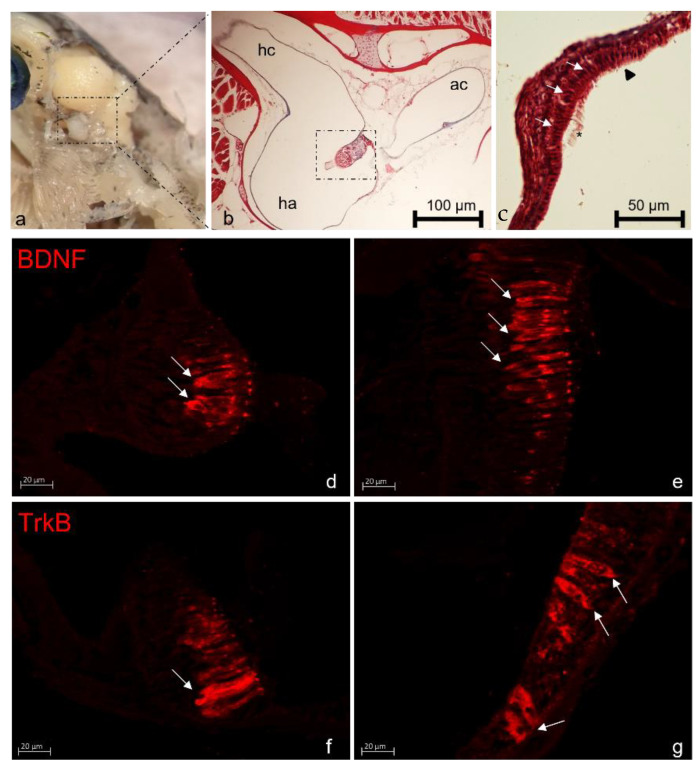
Stereomicrograph of zebrafish inner ear (**a**). Light micrograph of Masson Trichrome with aniline blue-stained dorso-ventral section of an adult zebrafish head; semicircular anterior canal (ac) and semicircular horizontal canal (hc) of the inner ear with its ampulla (ha) containing the crista ampullaris (dashed insert); (**b**). Utricular macula, sensory hair cells (white arrows) with numerous stereocilia (arrowheads) and less numerous but longer kinocilia (asterisk) are visible; (**c**). BDNF and TrkB immunoreactive sensory hair cells (arrows) in the crista ampullaris (**d**,**f**) and in the macula (**e**,**g**). Magnification 20× (**b**); 40× (**c**–**g**).

Furthermore, in order to perform a precise and correct cellular identification, the immunohistochemical procedures, using confocal laser microscopy, were performed on transgenic ET4 zebrafish larvae which express specific GFP in the cytoplasm of all hair cells [69] or using S100 protein as specific marker for hair cell identification [70,71,72]. During postnatal growth BDNF and TrkB mRNAs follow a parallel course, peaking at 20 dpf, thereafter progressively decreasing. At 20 and 30 dpf, immunoreactivity for BDNF and TrkB is specific and co-localized in all hairy cells of neuromasts; then, the number of immunoreactive cells decrease, and, by 180 dpf, BDNF just remains in a subpopulation of hairy cells, and TrkB in a small number of sensory and non-sensory cells. Therefore, in the lateral line system of zebrafish, the expression of BDNF and TrkB is parallel to age-related decline. Intriguingly, the BDNF and TrkB patterns of cell expression insinuate that autocrine/paracrine mechanisms might occur for this NT system within the neuromasts [73]. In addition to zebrafish, the distribution of immunoreactivity for BDNF-TrkB in the lateral line system, with a cell distribution almost entirely identical to that reported earlier, has been reported in the superficial and deep neuromasts of the alevins of Salmo salar and Salmo trutta [74], and TrkB has been also observed in the neuromast hair cells of Dicentrarchus labrax [75]. On the other hand, TrkB has not been detected in the sensory hair cells of the Nothobranchius guentheri neuromasts [76]. Although the role of BDNF-TrkB in the lateral line system is unknown, recent experiments indicate that the BDNF-TrkB axis regulates migration of the lateral line primordium influencing the expression of components of chemokine signaling, and the generation of progenitors of mechanoreceptors [77]. Presumably, BDNF-TrkB also participates in maintaining synaptic contacts between hair cells and afferent nerve fibers [78]. Zebrafish not only possess hair cells in the sensory lateral line system, but also in the inner ear. Hair cells in both organs are morphologically, functionally, and molecularly analogous to the inner ear hair cells of mammals. For this reason, recently, zebrafish have been widely used as an emerging animal model to analyze human sensorineural hearing loss [79]. To study inner ear physiology and diseases, zebrafish have contributed to those studies with the lateral line system. This primary mechanosensory system served as a model to study the mechanisms of hearing, deafness [80], and chemical or antibiotic-induced ototoxicity [81,82,83,84]. Moreover, this animal model, despite being compared to mammals, possesses the capacity to regenerate physiologically the hair cells damaged, restoring within a few days the mechanoreceptive functionality, thereby becoming a powerful model to investigate hair cells regeneration [85,86]. The inner ear in zebrafish consists of semicircular canals oriented following the three principal axes. These canals are filled with the endolymph and present, close to the end, a dilatated part within the sensory organs called the crista ampullaris. Moreover, the macule of the utricle also belongs to the inner ear structure, the saccule, and the lagena developing, respectively, balance, auditory, and a vestibular role. The sensory hair cells represent the principal cellular type identifiable in the macule together with the supporting and basal cells [87]. Very recently, the mRNA expression, as well as the protein occurrence for BDNF and its specific receptors TrkB in the zebrafish inner ear was performed using qRT-PCR and Western blot demonstrating the presence of both mRNA and protein at all ages examined from 8 dpf to adulthood. Moreover, using confocal laser microscopy, the localization of these proteins was demonstrated in the hair cells of the zebrafish inner ear during its whole life (Figure 2). Particularly, the immunolocalization for BDNF and TrkB was observed in the sensory patches of the macule of the utriculus, lagena, and sacculus as well as in the crista ampullaris of the semicircular canals [88].

These results are partially in agreement with a previous study performed on *Salmo salar* and *Salmo trutta* where the authors observed the localization of BDNF in the maculae of the utricle and saccule whereas it was absent from the sensory epithelium of the crista ampullaris [89]. The BDNF-TrkB system has been widely investigated in all vertebrates [90] including humans [52,91]. It is well known that the neurotrophins are involved in the development, as well as in the maintenance, of the inner ear in mammals, birds, and fish [90]. The decrease of the presence and expression of this growth factor family leads to a reduction of inner ear sensory cells and related nerve fibers which, however, can be partially improved with external BDNF administration [92,93,94].

## 3. BDNF-TrkB in the Retina

Regarding the visual system, the zebrafish eye and particularly the retina share morpho-functional and molecular features with human eye. It reveals visual function, even if it is not fully developed, by 72 hpf. Therefore, from this point of development, it is widely used to model human visual defects and understand the pathological molecular process of retinal degeneration [95,96,97]. Recently, using a genetics approach, this experimental animal model has become an intriguing organism to investigate eye congenital malformations such as microphthalmia, anophthalmia, and coloboma, which are considered among the principal causes of childhood blindness [98]. The zebrafish eye is interesting to perform drug screening and assess treatments based on BDNF delivery [99,100,101].

**Figure 3:**
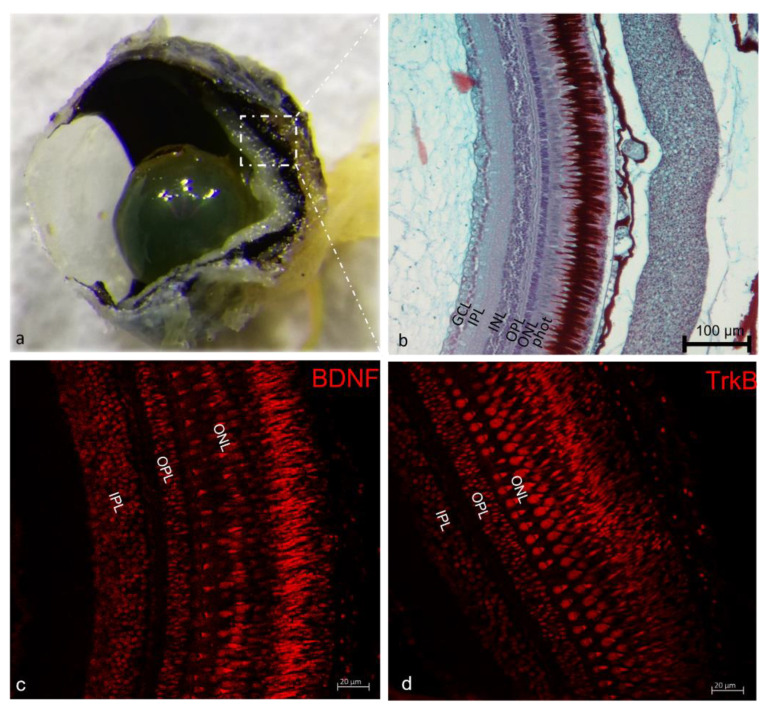
Stereomicrograph of zebrafish eye (**a**). Longitudinal section of zebrafish retina stained with Masson Trichrome with aniline blue, ganglion cell layer (GCL); inner plexiform layer (IPL), inner nuclear layer (INL), outer plexiform layer (OPL), outer nuclear layer (ONL), photoreceptor cell segment (phot); (**b**). Immunohistochemical localization of BDNF; (**c**) and TrkB; (**d**) in zebrafish retina layer. BDNF immunoreactivity was observed in the outer nuclear layer (ONL), outer plexiform layer (OPL) and inner plexiform layer (IPL), whereas TrkB immunoreactivity (**b**,**d**) was restricted to the inner plexiform layer (IPL). Magnification 20× (**b**); 40× (**c**,**d**). Magnification 20× (**d**), 40× (**c**,**d**).

The distribution of both BDNF and TrkB has been studied in the retina of marmoset monkeys, ferrets, rabbits, rats, mice, chickens, pigeons, barn owls, *Pseudemys turtles*, and *Xenopus frogs* [102]. This system is also present in different fish species, and it has been found in the retina of zebrafish, as well as in *Nothobranchius furzeri* [33], goldfish, carps [102], and the *Cichlasoma dimerus* retina [103]. Regarding this sensory organ, BDNF seems to have a role in maintaining the inner retinal integrity under normal conditions [104] and to participate in ganglion cell survival in culture [105]. BDNF act as a neuroprotective factor, particularly for retinal ganglion cells (RGCs). In zebrafish, BDNF and TrkB have been extensively found in the retina of zebrafish during development, in adults, and aged animals (Figure 3).

As a matter of fact, TrkB and BDNF mRNA are expressed through larval development and during adulthood, and both BDNF and TrkB were detected at the protein level [106]. In the zebrafish retina, the BDNF expression was detected in the ganglia cell layer (GCL), in the outer nuclear layer (ONL), the outer plexiform layer (OPL) and in the inner plexiform layer (IPL) (inner and outer sublayers of the IPL). In the GCL, it was found at early embryonic stages [16,107]. From 10 to 50 dph, all photoreceptors in the ONL express BDNF. Regarding the BDNF receptor, TrkB, its expression in the zebrafish retina is limited to fibers in the IPL, and very low levels of expression were found in the segments of the GCL from 10 to 50 dph. An intense TrkB immunostaining has been found in the outer sublayers of the IPL at 40 dpf and 50 dpf [108], and it was found to be localized in a diffuse and segmentary pattern in the GCL [17,73,102,109]. The BDNF/TrkB system has been detected in the retina of tench, specifically TrkB was observed in the INL and GCL and BDNF was found in the Müller cell processes and neuronal cells [110]. The expression of BDNF and TrkB in the zebrafish retina seems to be regulated by light. In fact, BDNF mRNA and BDNF immunostaining decrease after exposure to white-blue, blue, and white light, in an independent pattern of exposure. In return, the expression of TrkB mRNA was upregulated and TrkB immunostaining increased in the same experimental conditions. On the other hand, BDNF and TrkB mRNAs, and BDNF immunostaining decrease after exposure to darkness, although TrkB immunostaining remains unaffected. These variations demonstrate the action of the light on retinal BDNF and TrkB regulation in adult zebrafish. This could help to understand the pathophysiological mechanisms behind light-induced retinopathies [106]. Alterations in the balance of neurotrophic factors in the retina occur also in different pathogenesis mechanisms in humans. For instance, in patients with age-related macular degeneration, BDNF concentrations in the aqueous humor decrease and this correlates with a thickening of the outer retinal layer. The thinning of ONL may be a consequence of the inefficient protective effect of a low level of BDNF on photoreceptors [111]. Moreover, in patients with diabetes mellitus (DM), the serum and aqueous humor BDNF levels decreased [112]. In rat, the alterations of the balance of neurotrophic factors in the retina are implicated in developing age-related macular degeneration-like retinopathy. This finding is confirmed by the different expression patterns of BDNF between animals with progressive stages of retinopathy and healthy animals [113]. Indeed, in healthy rats mature BDNF (mBDNF) immunoreactivity was detected in the ganglion cells and in the Muller cells, while only the Muller cells displayed mBDNF protein in rats affected by retinopathy. Therefore, recently, researchers started to concentrate on the potential therapeutic role of neurotrophic factors in retinal diseases, neurodegeneration, and the possible involvement of glial and neuronal TrkB in neuroprotection and the neurotrophic factors; contribution to neurodegeneration [45]. Retinal disease treatment consisting of BDNF administration could help in the resolution of different pathologies caused by BDNF reduction. Recent studies have reported that NT intraocular delivery has been employed to treat different retinal pathologies [114,115,116]. In the retina of mice an increase of BDNF promotes retinal ganglion cell (RGC) survival after acute optic nerve injury. In the same way, axon survival is prolonged by BDNF upregulation [117]. In agreement with that, BDNF mimetics are able to restore retinal cell death and visual function, according to what Daly et al. [101] have demonstrated in a zebrafish model of inherited blindness [101]. Within the retina, the Sigma-1 receptor (S1R) may play a pivotal role in the modulation of BDNF levels. The treatment with S1R agonists can increase BDNF levels and might provide benefit in diseases such as glaucoma [118]. Increasing BDNF levels is effective in protecting retinal ganglion cell from damage and this could be reached by exogenous application of BDNF to the retina, increasing BDNF expression using viral vector systems and/or inducing BDNF expression by agents such as valproic acid.

## 4. BDNF-TrkB in the Taste Buds and Olfactory Mucosa

**Figure 4:**
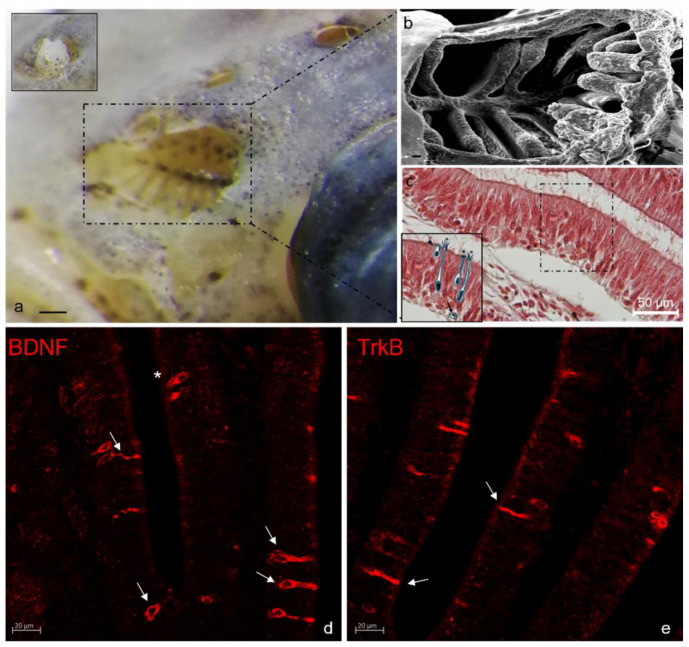
Stereomicrograph of zebrafish nose after removing nostril cartilage (**a**, insert); olfactory epithelium is visible (**a**, dashed area); Scanning electron micrograph of zebrafish olfactory epithelium organized in lamellae (**b**); Sensory cells (insert) of the olfactory epithelium of the sensory part of the rosette lamella: cryptic neuron (arrowhead), neuron with microvilli (star), ciliate neurons (asterisks), immature neuron (arrow) (**c**); Immunohistochemical localization of BDNF (**d**); and TrkB (**e**) in crypt cells (asterisks) and sensory cells (arrows) in basal and sensory segment of olfactory lamellae. Scale bar: 1 mm (**a**), 20 µm (**b**). Magnification 40× (**c**–**e**).

**Figure 5:**
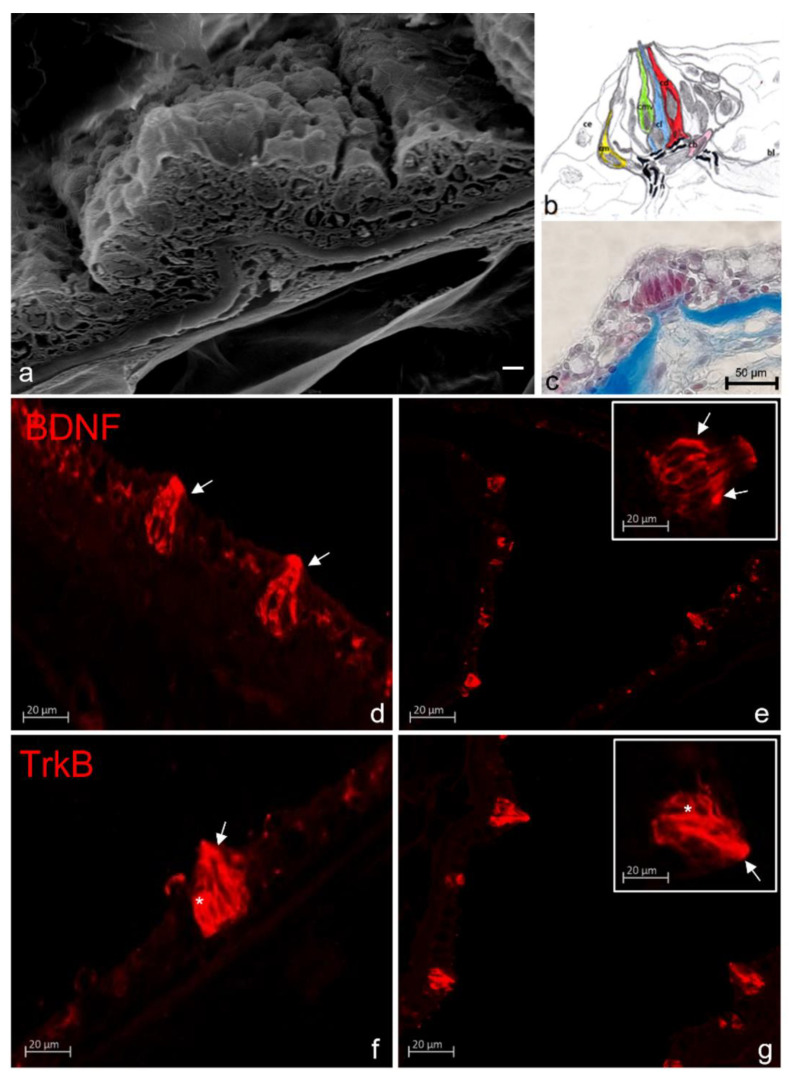
Scanning electron micrograph of zebrafish taste bud (**a**); Schematic drawing of a taste bud (**b**) with light cells (cl), dark cells (cd), light cells with microvilli (cmv), marginal cells (cm), basal cells (cb), nerve (n), and basal layer (bl). Light micrograph of a Masson Trichrome with aniline, blue-stained sagittal section of a taste bud from the oral cavity (**c**); BDNF immunoreactive dark cells (asterisks) in zebrafish cutaneous (**d**); and oral (**e**) taste buds. TrkB immunoreactive dark cells (arrows) and light cells (asterisks) in zebrafish cutaneous (**f**); and oral (**g**) taste buds. Scale bar: 20 µm (**a**). Magnification 40× (**c**–**g**).

Another fundamental part of the complex mechanism of sensation in zebrafish is constituted from the chemosensory system with particular attention to the olfactory organs and taste buds, that exert a pivotal function in many behavior aspects related to the food intake and sexual partner localization [119]. Some study has been performed to analyze the interconnection network among the different sensory and non-sensory cell types, forming the olfactory organs, that are at the basis of the regeneration or repair process involved in olfactory [120] and taste human disorders [121]. In this field, only a few studies have been conducted to demonstrate the involvement of neurotrophins and its receptor in the fish olfactory system [122] and in taste [74,75,122,123]. It is well known that BDNF is involved in the biology of chemosensory systems. BDNF is one of the growth factors involved in the generation and differentiation of new olfactory neurons [124,125,126], expressed in both the olfactory bulb and the olfactory epithelium during the regeneration process. Moreover, the presence of its receptor TrkB in mature olfactory neurons shows that BDNF is needed during the regeneration process of olfactory neurons [61]. BDNF plays a role in the continuous cell regeneration and the turnover of the so-called “rosette” (Figure 4) and taste buds (Figure 5) in young and adult teleosts.

In zebrafish, Trk receptors were identified in two protein bands corresponding to the full-length isoforms of TrkA (140 kDa) and TrkB (145 kDa) and have been also localized in the same taste organs. Unfortunately, it was impossible to demonstrate that the two receptors colocalized in the same cellular type [71]. Taste buds of adult zebrafish express TrkA- and TrkB-like [123] as in mammals [127,128,129], and in *D. labrax* [19]. In the rosette, in adult zebrafish, ciliated cells in the sensory segment of olfactory lamellae have shown BDNF immunoreactivity. In the olfactory epithelium of zebrafish in the larval stage immunopositivity for pro BDNF, the precursor of mature BDNF, has been observed. In particular, it was shown in microvillous cells and in ciliated cells where it colocalizes with two different calcium-binding proteins, respectively, S100 and Calretinin N-18. The sensory cells of adult zebrafish taste buds have been immunoreactive to BDNF and Calretinin N-18. Colocalization for these proteins has been demonstrated both in taste buds’ light cells and in dark cells. Moreover, BDNF and Calretinin N-18 colocalize in some Merkel-like basal cells. These results are comparable in both skin and oral taste buds.

## 5. Material and Methods

### 5.1. Zebrafish Breeding and Tissue Treatments

In this study adult zebrafish, kept on a 14 h day, 10 h night cycle at a constant temperature of 28.5 °C and fed twice a day, were used. The fish were sacrificed with a lethal dose of tricaine methane sulfonate (MS222; 1000–10,000 mg L^−1^). The samples were quickly removed, fixed in 4% paraformaldehyde in phosphate buffered saline (PBS) 0.1 m (pH = 7.4) for 12–18 h, dehydrated trough graded ethanol series, clarified in xylene, for paraffin wax embedding. Included tissues were then cut in to 7 µm thick serial sections and collected on gelatin-coated microscope slides and then routinely processed for histological staining with hematoxylin-eosin (Carazzi’s hematoxylin nuclear staining, 05-M06012; eosin Y 1% acqueous solution cytoplasmic staining, 05-M10002, Bio-optica Milano s.p.a) and Masson Trichrome with aniline blue method (04-010802, Bio-Optica Milano s.p.a.). Sections were examined under a Leica DMRB light microscope. Then, serial sections were processed for immunofluorescence analysis. The experimental protocols, employed in this study, were in accordance with the principles outlined in the Declaration of Helsinki and the samples used in this review coming from a previous experimentation approved by Italian Ministry of Health (A.M. n. 50, 8 August 2013).

### 5.2. Localization of BDNF/TrkB in Sensory Organs of Zebrafish

To analyze the expression of BDNF/TrkB system in the sensory organs the sections were deparaffinized and rehydrated, washed in Phosphate-Buffered Saline (PBS) 0.1 M pH = 7.4, and incubated in 0.3% H_2_O_2_ (PBS) solution for 3 min to prevent the activity of endogenous peroxidase; then fetal bovine serum was added to rinsed sections. Sections were incubated overnight at 4 °C in a humid chamber with polyclonal antibodies anti- BDNF (Merck Millipore, Darmstadt, Germany; AB1534SP; diluted 1:100) and with anti-TrkB (Santa Cruz Biotechnology, Inc. CA, USA, sc-12, diluted 1:100). After rinsing in PBS, the sections were incubated for 1 h with anti-Rabbit Secondary Antibody, Alexa Fluor 594 (Invitrogen, Waltham, MA, USA; A-11012, diluted 1:300). Finally washed, dehydrated, and mounted with Fluoromount Aqueous Mounting Medium (Sigma Aldrich, St. Louis, MO, USA). The sections were analyzed, and images acquired using a Zeiss LSMDUO confocal laser scanning microscope with META module (Carl Zeiss Micro Imaging, Oberkochen, Germany) microscope LSM700 Axio Observer. Zen 2011 (LSM 700 Zeiss software). Each image was rapidly acquired to minimize photodegradation.

### 5.3. Scanning Electron Microscopy Analysis

Samples were fixed in 2.5% glutaraldehyde in Sörensen phosphate buffer 0.1 M. After several rinsing steps in the same buffer, they were dehydrated in a graded alcohols series, critical-point dried in a Balzers CPD 030 (BAL-TEC AG, Balzers, Liechtenstein), sputter coated with 3 nm gold in a SCD 050 sample coater (BAL-TEC AG, Balzers, Liechtenstein) and examined under a Zeiss EVO LS 10 (Carl Zeiss NTS, Oberkochen, Germany).

## 6. Conclusions

Evidence gathered in this review confirms the pivotal role of the BDNF/TrkB system in the physiopathology of sensory cells of the sensory systems and their possible interaction with calcium-binding proteins. The involvement of the BDNF-TrkB axis in the biology of sensory systems is demonstrated by the studies conducted on zebrafish which is emerging as an increasingly successful experimental model for studying hearing and balance, retinal diseases, and odor detection. In particular, it is a model of choice for investigating the regeneration processes that concern sensory systems and that seem to be regulated by neurotrophins and their signal-transducing receptors. Further studies, considering the use of BDNF and TrkB transgenic models, are needed to analyze the functional activity of BDNF in the chemo- and mechanosensory organs of zebrafish. This could help to clarify some neurotrophins’ unknown functions on the sensory cells of taste buds and to find possible therapeutic applications of these growth factors to treat human diseases.

## Data Availability

All data presented this study are available from the corresponding author, upon responsible request.
